# Complete Genome Sequence of a Highly Pathogenic Porcine Reproductive and Respiratory Syndrome Virus Variant with a New Insertion in Glycoprotein 5, Isolated from a Stillborn Fetus

**DOI:** 10.1128/genomeA.01359-15

**Published:** 2015-11-25

**Authors:** Can Liu, Lei Zhang, Yi-bao Ning

**Affiliations:** a China Institute of Veterinary Drug Control, Beijing, China; b China Agricultural University, Beijing, China

## Abstract

Here, we report the complete genome sequence of strain GD-2011, a highly pathogenic porcine reproductive and respiratory syndrome virus (HP-PRRSV) that was isolated from a stillborn fetus. The GD-2011 strain is characterized by a discontinuous 30–amino acid deletion in the nonstructural protein 2. In addition, GD-2011 had a 1–amino acid insertion in glycoprotein 5, which does not exist in any other HP-PRRSV strains.

## GENOME ANNOUNCEMENT

Porcine reproductive and respiratory syndrome (PRRS) has engendered immense economic losses to the swine industry worldwide since it emerged in the late 1980s (1–4). PRRS virus (PRRSV), the etiologic agent that belongs to the family *Arteriviridae* in the order *Nidovirales*, is a single-stranded positive-sense RNA virus. The first Chinese PRRSV strain, CH-1a, was isolated in 1996. Then, the highly pathogenic PRRSV (HP-PRRSV) variant JXA1, characterized by a discontinuous 30–amino acid (aa) deletion in its NSP2-coding region was isolated in 2006. Both CH-1a and JXA1 are a North American–type PRRSV (1, 3). The first isolated European-type PRRSV strain was reported in 2011 (5). The PRRSV epidemics in China are very intricate, and HP-PRRSVs have remained prominent since 2006 (6–9), and HP-PRRSV isolates from 2007 to 2010 have similar pathogenicity to the 2006 isolate JXA1 (10). Furthermore, PRRSV has shown remarkable genetic variation through mutation or recombination, resulting in the emergence of novel variants (11). Here, we report the complete genome sequence of the HP-PRRSV variant GD-2011.

The GD-2011 strain was isolated from the lung of a stillborn fetus in a farrow-to-finish, unvaccinated farm in Guangdong province, southern China. This farm suffered a grievous outbreak of PRRS in May 2011 with nearly 53% morbidity and 36% mortality. To determine its complete genome sequence, 14 pairs of specific primers were applied to amplify 14 overlapped fragments of the GD-2011 genome by reverse transcription (RT)-PCR. The PCR products were purified, cloned into a pMD18-T vector (TaKaRa, China), sequenced three times with an ABI3730XL genome sequencer, and assembled into the full-length sequence with SeqMan software (DNAStar Inc.). Excluding the poly(A) tail, the genomic sequence of GD-2011 was 15,323 nucleotides in length. GD-2011 shared 89.4%, 61.7%, and 99.2% nucleotide identity with the NA prototype VR-2332, the European prototype Lelystad virus, and the representative HP-PRRSV strain JXA1, respectively. Genetic analysis revealed that GD-2011 shared 97.01 to 98.78% identity with the representative HP-PRRSV strains (JXA1, HuN4, and GD) and 51.20% identity with the representative European PRRSV strain (Lelystad), and exhibited two unique discontinuous deletions of 30-aa deletion within NSP2-coding region, just as JXA1. In addition, compared with 55 HP-PRRSV strains, it is worth noting that a 1-aa insertion in the GP5 region of GD-2011 was found. Phylogenetic analysis illustrated that GD-2011 belongs to the same subgroup as other highly pathogenic PRRSV strains, and its homology with HP-PRRSV strains of 2010 is higher than those from 2006 to 2009. The genome data of GD-2011 will be helpful for understanding the epidemiology, evolution, and pathogenesis of PRRSV in pigs.

(Parts of the present results were presented at the 2013 International Porcine Reproductive and Respiratory Syndrome Symposium, Beijing, China, 20 to 22 May 2013.) 

### Nucleotide sequence accession number.

The complete genome sequence of strain GD-2011 is available in GenBank under the accession number KC527830.
